# Giant splenic artery aneurysm rupture into the stomach that was successfully managed with emergency distal pancreatectomy

**DOI:** 10.1186/s40792-022-01498-3

**Published:** 2022-08-02

**Authors:** Chihiro Yoshikawa, Ichiro Yamato, Yasuyuki Nakata, Tadashi Nakagawa, Takashi Inoue, Mitsuhiro Nakatani, Daiki Nezu, Shunsuke Doi, Yasuhiro Kuroda, Kazuki Fujii, Shouhei Kishida, Midori Kamikubo, Saiho Ko

**Affiliations:** Department of Surgery, Nara Prefecture General Medical Center, 2-897-5 Shichijo-Nishimachi, Nara, 630-8581 Japan

**Keywords:** Splenic artery aneurysm, Upper gastrointestinal bleeding, Hemorrhagic shock, Emergency surgery, Case report

## Abstract

**Background:**

Splenic artery aneurysms usually rupture into the free peritoneal space and rarely into the gastrointestinal tract. We report the case of a patient with a giant splenic artery aneurysm that ruptured in to the stomach with hemorrhagic shock and was successfully treated with emergency surgery.

**Case presentation:**

A 59-year-old man presented to the emergency department with chest pain and syncope. Contrast-enhanced computed tomography showed splenic artery aneurysm with active contrast extravasation. He developed upper gastrointestinal (UGI) bleeding and hypovolemic shock. We diagnosed a splenic artery aneurysm ruptured in to the stomach, performed emergency distal splenopancreatectomy including the aneurysm and partial gastric resection, and could prevent patient death.

**Conclusions:**

This report shows that splenic artery aneurysm can cause UGI bleeding. Thus, clinicians should be alert about this condition when managing patients with UGI bleeding and/or splenic artery aneurysm.

## Background

The rupture of a splenic artery aneurysm leads to massive life-threatening bleeding with hemodynamic instability, usually into the free peritoneal space and more rarely into the gastrointestinal tract. We report the case of a patient with giant splenic artery aneurysm that ruptured in to the stomach with hemorrhagic shock and was successfully treated with emergency surgery.

## Case presentation

A 59-year-old man with no related medical history presented to the emergency room with chest pain and syncope. On admission, he showed signs of hypovolemic shock with paleness, sweating, low blood pressure (BP) of 62/36 mmHg, poor peripheral perfusion, and acutely deteriorating anemia (blood level of 12.3 d/dL on first check and 9.5 d/dL on the second) check; he was alert at this time.

**Fig. 1:**
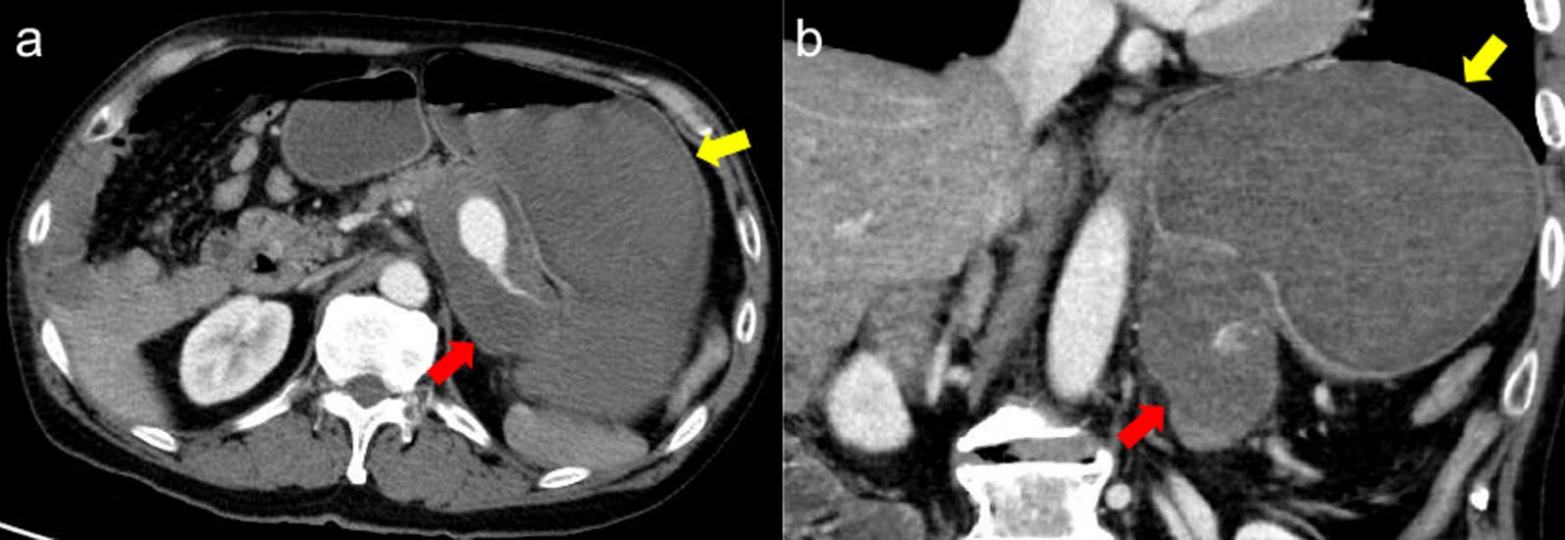
Abdominal contrast-enhanced computed tomography findings. **a** Splenic artery aneurysm (SAA) (10 cm in maximum diameter) with extravasation of contrast from the splenic artery. The red arrow indicates the SAA, and the yellow arrow indicates the stomach. **b** SAA in close communication with the posterior wall of the stomach. The red arrow indicates the SAA, and the yellow arrow indicates the stomach

**Fig. 2:**
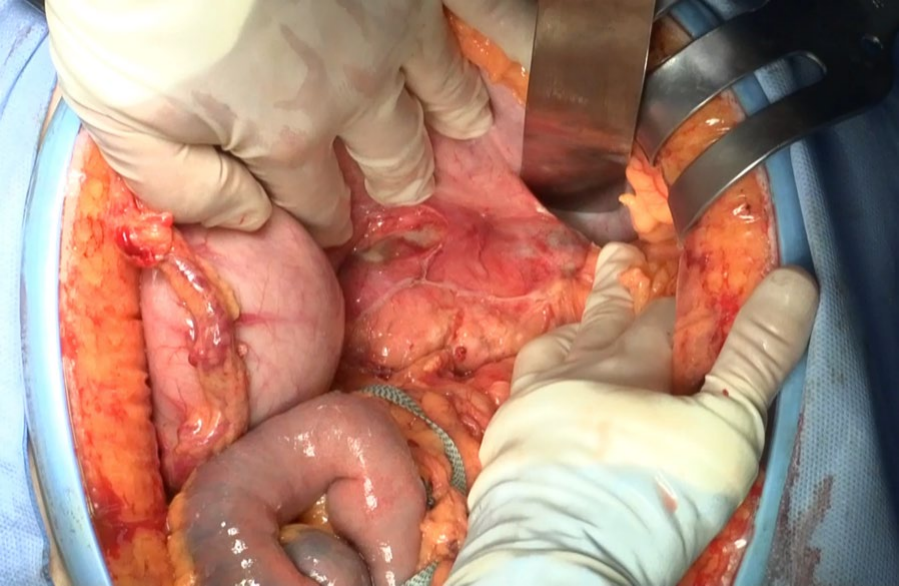
Intraoperative findings. A splenic artery aneurysm wall densely adhered to the posterior wall of the stomach

**Fig. 3:**
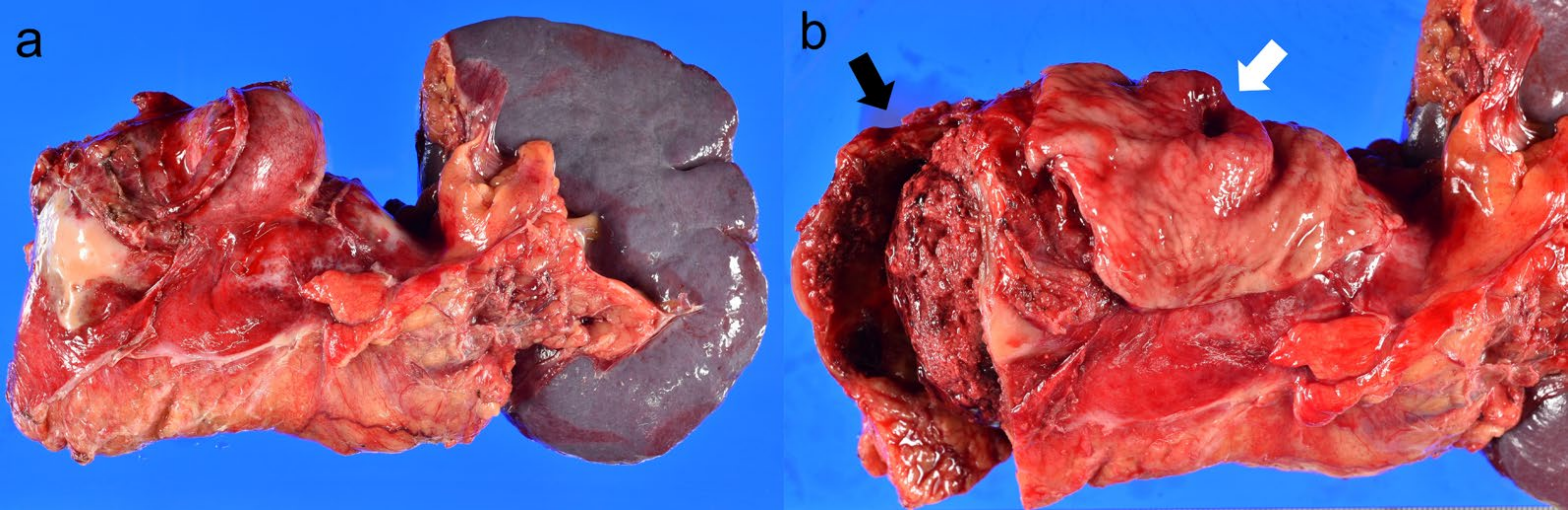
Macroscopic findings. **a** Splenic artery aneurysm (SAA) with body–tail of the pancreas and the spleen. A part of the stomach wall was resected with the SAA. **b** The lumen of the splenic artery aneurysm (black arrow) Point of communication with the stomach (white arrow)

Electrocardiography and chest radiography showed no significant findings. After fluid resuscitation, contrast-enhanced computed tomography (CT) showed that the middle and distal third of the splenic artery were fully replaced by an aneurysm (10 cm in maximum diameter), with active contrast extravasation into the aneurysm. It was packed into the posterior wall of the stomach. The stomach showed significant expansion with mud fluid (Fig. 1). Subsequently, we suspected a splenic artery pseudoaneurysm that had ruptured into the stomach. A nasogastric tube was inserted to aid in the prevention of vomiting and aspiration because we suspected UGI bleeding. Bright red blood (300 mL) was drawn using the tube. At that time, he lost consciousness showing a rapid drop in BP of 54/37 mmHg, HR70 again. In total, 4 units of red blood cells (RBCs) and 2 units of fresh frozen plasma (FFP) were administered, and while preparing to move the patient to the operating room, the systolic BP stabilized to > 100 mmHg. Laparotomy was performed immediately. The aneurysm was located in the distal part of the splenic artery, adhering to the body of the pancreas. Its wall densely adhered to the posterior wall of the stomach covered with necrotic slough around that was the ruptured root of the splenic aneurysm to the stomach (Fig. 2). The pancreas was mobilized along with the splenic artery from the retroperitoneum, and a tape was passed around the body of the pancreas to enable bleeding control and the ligation of the splenic artery. After dissecting the body–tail of the pancreas, we performed a distal splenopancreatectomy including the aneurysm and partial gastric resection (Fig. 3). The total durations required for the surgery and general anesthesia were 212 min and 292 min, respectively. The intraoperative fluid balance was + 440 mL with an estimated blood loss of 1950 mL, including gastric clots and the urine output of 340 mL. Intraoperatively, 6 units of RBCs and 2 units of FFP were transfused.

The postoperative vitals of the patient stabilized immediately and his general condition improved quickly. He was discharged from the hospital on the 42nd postoperative day, after waiting for decrease in the pancreatic fistula output and the removal of the drainage tube. The pathology of the resected specimen showed a splenic artery aneurysmal sac eroding into the gastric mucosa. The surrounding gastric mucosa did not show any malignant evidence, but exhibited the destruction of the elastic fibers of the vessel wall, suggesting pseudoaneurysm of the splenic artery.

## Discussion

Splenic artery aneurysm (SAA) is the third most common abdominal vessel aneurysm after abdominal aortic aneurysm and iliac artery aneurysm. In addition, it is the most common visceral vessel aneurysm. With the increasing availability of imaging examinations, there has been a rise in the reported cases of SAA [1, 2]. The major complication of SAA was rupture. The risk of rupture for a true aneurysm is very low (2–3%); however, this risk substantially increases for pseudoaneurysms (37–47%) with a 90% mortality rate [3]. Their rupture leads to massive life-threatening bleeding with hemodynamic instability.

Liu et al. reported that SAAs usually rupture into the peritoneal cavity, and < 30% of them perforate into the lumen of intra-abdominal visceral organs [4]. Shibuta reported that the percentage of perforation to the peritoneal cavity, pancreatic duct, stomach, colon, retroperitoneum, and pancreatic cyst was 51.0%, 21.6%, 9.8%, 9.8%, 3.9%, and 3.9%, respectively [5]. Thus, the perforation of SAAs in the stomach is rare.

In case of non-ruptured SAAs, the patient is asymptomatic. They are most commonly diagnosed during examination for other reasons. If their vital signs are stable with no signs of bleeding, the lesion must be evaluated using contrast-enhanced computed tomography and selective angiography. In such cases, the following treatments are recommended: percutaneous, intravascular embolization, and stenting or laparoscopic ligation of the aneurysm [1, 2, 6, 6–8]. In the case of a ruptured SAA, the patient had upper abdominal pain and presented with hematemesis or hematochezia, with hypovolemic shock.

Our patient experienced rupture with massive intragastric bleeding and syncope because of hypovolemic shock. Contrast-enhanced CT of his abdomen detected a SAA with intragastric bleeding. If the bleeding point is not identified, hemostasis during laparotomy is very challenging. However, in our case, the bleeding site was detected, and we could perform laparotomy.

In contrast, some studies have reported the success rates from 75 to 85% following interventional radiology (IVR) [11, 12]. Recently, IVR has been recognized as a first-choice of treatment for SAA. IVR is chosen because it is minimally invasive, can be performed in patients with poor general condition, such as in those with hemorrhagic shock, and can be performed in patients with serious conditions that pose a high risk of general anesthesia—similar to this case. The angiographic treatment might be associated with other severe complications, including the formation of abscesses in the spleen and embolism in the arterial system [8]. However, even if a patient suffered such complications, surgery could be performed after the condition was stabilized by IVR.

Angiography might not be a definitive treatment in patients with aneurysm and gastric bleeding who have a fistulous connection. Moreover, there have been several reports of post-procedural coil migration and coils extruding into the gastric lumen in such cases [9, 10]. However, in this regard, given that the aneurysm was located in the distal splenic artery in the present case, temporary hemostasis was likely to be achieved with coiling in the proximal splenic artery, based on the intraoperative findings.

Because of insufficient time, we could not wait for the radiologist to arrive; therefore, we opted for surgery in this case. Although IVR is the first-choice of treatment for splenic aneurysms, emergency surgery may be an option when the aneurysm is large and perforates the gastrointestinal tract and there is a risk of coil dislodgement or when the vitals are unstable and one cannot wait for the radiologist to arrive, as in this case.

## Conclusion

We presented the case of a patient with a giant SAA that ruptured into the stomach with hemorrhagic shock and was successfully treated with immediate surgery. Aneurysm rupture to the stomach is a rare case of massive UGI bleeding. It is crucial to be aware of this condition to make a timely treatment decision that is critical for saving the patient’s life.

## Data Availability

No applicable.

## Abbreviations

UGI: Upper gastrointestinal
SAA: Splenic artery aneurysm
