# Correction: Giant splenic artery aneurysm rupture into the stomach that was successfully managed with emergency distal pancreatectomy

**DOI:** 10.1186/s40792-022-01577-5

**Published:** 2022-12-08

**Authors:** Chihiro Yoshikawa, Ichiro Yamato, Yasuyuki Nakata, Tadashi Nakagawa, Takashi Inoue, Mitsuhiro Nakatani, Daiki Nezu, Shunsuke Doi, Yasuhiro Kuroda, Kazuki Fujii, Shouhei Kishida, Midori Kamikubo, Saiho Ko

**Affiliations:** Department of Surgery, Nara Prefecture General Medical Center, 2-897-5 Shichijo-Nishimachi, Nara, 630-8581 Japan

**Correction: Surgical Case Reports (2022) 8(1):148** **https://doi.org/10.1186/s40792-022-01498-3**

Following publication of the original article [1], the authors reported an error in two of the author names.

Author name “Syunsuke Doi” was mistakenly spelled, the correct author name should read “Shunsuke Doi”.

Author name “Syouhei Kishida” was mistakenly spelled, the correct author name should read “Shouhei Kishida”.

The original article [1] has been updated.
